# Serum estrone concentration, estrone sulfate/estrone ratio and BMI are associated with human epidermal growth factor receptor 2 and progesterone receptor status in postmenopausal primary breast cancer patients suffering invasive ductal carcinoma

**DOI:** 10.1186/s40064-015-1171-8

**Published:** 2015-07-31

**Authors:** Borbála Vincze, Bence Kapuvári, Nóra Udvarhelyi, Zsolt Horváth, Zoltán Mátrai, Ferenc Czeyda-Pommersheim, Krisztina Kőhalmy, Judit Kovács, Mariann Boldizsár, István Láng, Miklós Kásler

**Affiliations:** Department of Biochemistry, National Institute of Oncology, 1122 Budapest, Ráth György u. 7-9., Hungary; Surgical and Molecular Tumor Pathology Centre, National Institute of Oncology, Budapest, Hungary; Clinic of Oncology, Centre of Clinics, University of Debrecen, 4032 Debrecen, Nagyerdei krt. 98., Hungary; Department of General and Thoracic Surgery, National Institute of Oncology, Budapest, Hungary; Medical Oncology and Clinical Pharmacology “B”, National Institute of Oncology, Budapest, Hungary; National Institute of Oncology, Budapest, Hungary

**Keywords:** Postmenopausal breast cancer, Invasive ductal carcinoma, Estrone, Estrone sulfate, Progesterone receptor, HER2

## Abstract

**Background:**

We investigated in postmenopausal women with primary breast cancer prior to surgical intervention whether, serum levels of different steroid hormones and hormonal precursors associated with tumor tissue estrogen receptor (ER), progesterone receptor (PR) and human epidermal growth factor receptor 2 (HER2) status.

**Methods:**

We enrolled 1,042 patients suffering invasive ductal carcinoma undergoing surgical resection in the National Institute of Oncology, Hungary between 2003 and 2011. Serum parameters were measured by RIA/IRMA assays; tumor tissue ER, PR and HER2 status was assessed histologically. Patients were classified according to tumor receptor status. Case–case analysis subjects were categorized into four subgroups based on serum hormone concentrations in ER, PR and HER2 receptor-negative cases, respectively.

**Results:**

Serum estrone sulfate and dehydroepiandrosterone sulfate levels correlated with each other and also with serum estrone and estradiol levels. According to case–case study the odds ratios in the highest quartile were 1.517 (p = 0.0305, P_trend_ = 0.0394) for androstenedione, 1.495 (p = 0.0317, P_trend_ < 0.0105) for estrone and 0.654 (p = 0.0273, P_trend_ < 0.0151) for estrone sulfate/estrone ratio in PR+ vs. PR− tumors. Regarding HER2 status (HER2+ vs. HER2−), the odds ratios for estrone, estrone sulfate and estrone sulfate/estrone ratio were 0.530 (p = 0.0234, P_trend_ = 0.0595), 2.438 (p = 0.0042, P_trend_ < 0.0066) and 3.118 (p = 0.0001, P_trend_ < 0.0001) in the highest quartile, respectively. Of note significantly increased BMI associates with PR+ and ER +/PR+ status while significantly decreased BMI was observed in HER2+ cases.

**Conclusions:**

Taken together, measurement of serum estrone and estrone sulfate concentrations prior to surgical intervention might support the individualization of regime in postmenopausal primary breast cancer patients.

## Background

Breast cancer is a very heterogeneous disease that can be classified to molecular, histopathologic and clinical subgroups. Anticancer therapy is determined by biological characteristics and stage of the tumor. Most important biological features determining therapy are endocrine sensitivity, human epidermal growth factor receptor 2 (HER2) expression and proliferative capability of the tumor (Láng et al. 2012).

Approximately 70% of breast cancer cases express estrogen receptor (ER) and progesterone receptor (PR) thus are referred as hormone receptor (HR)-positive. HER2 expression is presented in 20–25% of breast cancer cases that are classified as HER2-positive (HER2+) (Ross et al. 2009; Dawood et al. 2010). Approximately 50% of HER2-positive cases are also HR-positive (ER+/PR+/HER2+) (Dowsett et al. 2008; Tripathy et al. 2013; Mehta and Tripathy 2014). About 10–20% of invasive breast cancer cases do not express ER, PR or HER2 and are termed as of triple negative receptor status (ER−/PR−/HER2−) (Perou 2011; Aysola et al. 2013).

Estrogens play a crucial role in breast cancer progression through inhibition of apoptosis and stimulation of cell proliferation via ER activation (Hankinson and Eliassen 2007). Several epidemiological studies indicate that plasma estradiol (E2), adrenal androgens and testosterone (TE) levels were higher in women who developed ER-positive breast cancer later (Key et al. 2002; Zeleniuch-Jacquotte et al. 2004; Missmer et al. 2004; Kaaks et al. 2005; Cummings et al. 2005; Sieri et al. 2009; Endogenous Hormones and Breast Cancer Collaborative Group 2013). A Danish population-based prospective study revealed that the association between the risk of postmenopausal breast cancer and serum estrone (E1) or estrone sulfate (E1S) levels is stronger than that between E2 and breast cancer risk (Würtz et al. 2012). Zhang et al. (2013) demonstrated that blood sex hormone levels measured at a single time-point would predict the development of breast cancer within up 16–20 years.

Epidemiological studies focused on the association between sex hormones and breast cancers or excluded ER-negative breast cancers from the analysis (Key et al. 2002; Zeleniuch-Jacquotte et al. 2004; Kaaks et al. 2005; Cummings et al. 2005). The reason of exclusions from several cohort studies is that the number of ER−/PR− cases was relatively small; the statistical power was insufficient to assess other relevant breast cancer subtypes, such as triple negative, or HER2-positive (Zhang et al. 2013).

It is well documented that ER/PR and HER2 status predict the clinical outcome and the response to adjuvant endocrine therapy or poly-chemotherapy. However, a detailed hormone profile determined before surgical intervention can also support to predict the hormone sensitivity of the tumor. Based on biological and clinical observations it was suggested that plasma levels of sulfoconjugated and unconjugated steroid hormones and tissue-specific expression of steroid sulfatase (STS) might play a significant role in breast cancer biology and might regulate the effects of endocrine therapy (Falany and Falany 2007). Kim et al. found that high preoperative serum E2 level indicate worse prognosis in postmenopausal women with breast cancer, particularly in those with ER-negative cancer (Kim et al. 2013). However, the interaction between sexual hormone levels before surgery and receptor status was not investigated widely.

Our aim was to investigate whether circulating steroid hormone levels including sexual hormones and their precursors along with sex hormone binding globulin [i.e. E1, E1S, E2, TE, dehydroepiandrosterone (DHEA), DHEA sulfate (DHEAS), androstenedione (AD) and SHBG] measured prior surgical intervention show any association with tumor ER, PR and HER2 status in postmenopausal women with primary breast cancer. In our case–case study the relationship between serum sexual hormone levels and tumor ER, PR and HER2 status was retrospectively studied using data collected from postmenopausal patients treated with breast cancer between 2003 and 2011 in the National Institute of Oncology, Hungary.

## Methods

### Patients

Our study involved 1381 postmenopausal patients with primary breast cancer (stages ranging between 0 and III). Women were considered postmenopausal when they reported not having any menstrual cycles in the past 24 months; those with bilateral ovariectomy in medical history; and those with age above 55 years (Kaaks et al. 2005).

Distribution of the patients according to age: 485 cases (55–59 years), 528 cases (60–69 years), 271 cases (70–79 years) and 97 cases (≥80 years). The mean age of the population studied was 64.7 ± 9.1 years.

The diagnosis of breast cancer was confirmed by histology in all cases. Tumors had been diagnosed by experienced pathologists using standard criteria for histology and grading. All patients had resectable stage 0–III tumors according to the TNM 6.0 staging (Union International Cancer Congress, TisN0M0-T2N3M0). The histological diagnosis was mainly invasive ductal carcinoma (IDC) 1,042 (75.45%), IDC in situ (DCIS) 84 (6.08%), invasive lobular carcinoma (ILC) 140 (10.14%) and others 115 (8.33%) (including metaplastic, adenoid, papillary, apocrine, cribriform, medullary, mucinous and tubular invasive carcinomas). Patients were diagnosed mainly with IDC (1,042 cases) therefore statistical analysis was performed within this subgroup.

The study was approved by the Institutional Review Board and Ethics Committee of National Institute of Oncology, Hungary since 2003. Permission of the Hungarian Regional Committee of Science and Research Ethics was obtained for retrospective evaluation of the data (Number of permission: 322/2014).

At the time of blood sampling all patients was informed about the purpose of our study. Written informed consent was obtained from all individual participants included in the study. Patients donated 8–10 ml blood sample and completed a questionnaire about reproductive history, previous use of contraceptives, postmenopausal hormone replacement therapy (HRT). Women who used HRT at the last 6 months and/or had a diagnosis of cancer within 10 years before surgical intervention were not elected to the present study. Patients who received neoadjuvant chemotherapy were excluded, as well.

### Determination of serum hormone levels

Blood samples were collected according to a standardized protocol. Briefly, the whole blood was centrifuged at 2,500 *g* for 15 min. The serum was removed from the blood clot and stored in aliquots at −20°C until the determination. Measurements were carried out within 2–3 months in all cases. Steroid hormone assays were performed in our Laboratory on Department of Biochemistry (NIO). Since the concentration of serum E2 is usually lower than 40 pmol l^−1^ in postmenopausal women, serum E2 level was determined by using an ultra-sensitive estradiol radioimmunoassay (RIA) kit (Immunotech, Prague, Czech Republic, detection limit: 8.14 pmol l^−1^). E1S, DHEA and AD were measured by RIA kit (Immunotech, Prague, Czech Republic). Total TE and DHEAS were measured by Immunotech SAS RIA kit (Marseille, France); E1 concentration was measured by RIA with a Diasource Immunoassays S.A. kit (Louvain-la-Neuve, Belgium). SHBG was measured by Izinta kit (Isotope Institute, Budapest, Hungary). The mean intra- and inter-assay coefficient of variations were 7.5 and 9.4% for E2, 14.8 and 15.0% for TE, 6.3 and 8.6% for E1, 6.1 and 4.3% for SHBG, 9.2 and 8.8% for E1S, 5.6 and 9.8% for AD, 7.4 and 10.6% for DHEAS, 3.8 and 8.6% for DHEA. Stratec SR 300 (Birkenfeld, Germany) an automatic, open analyser system was used to detect ^125^I radioactivity.

Free TE and E2 concentrations were calculated from the concentrations of E2, TE and SHBG according to previous studies (Vermeulen et al. 1999; Endogenous Hormones and Breast Cancer Collaborative Group 2003).

### Assessment of tumor receptor status

The ER and PR status were evaluated histologically on immunohistochemical (IHC) slides [ER: SP1 (NeoMarkers), PR: NCL-L PGR-312 (Novocastra)]. ER and PR positivity were defined when at least 10% prevalence of malignant cells exhibiting staining characteristics.

HER2 protein overexpression was assessed by IHC method using three different antibodies: RTV-CB11 (Novocastra), C-erbB-2/Her-2/neu SP3 clone (NeoMarkers) and HercepTest (DAKO). Samples were scored using the recommended scoring system for the HercepTest.

HER2 gene amplification was tested with the Inform-HER2 test by Ventana. The updated cut-off value for positive cases is more than six copies of the gene. HER2 was scored positive if the result was 3+; 2+ was considered to be positive only if it was confirmed by fluorescence in situ hybridization (FISH).

### Statistical analysis

MedCalc Software was used for statistical analysis. In the case of a normal distribution, the correlation between serum hormone parameters was calculated using the correlation coefficient (r), in the remaining cases Spearman’s coefficient of rank correlation (rho) was used. 95% confidence interval (95% CI) for r or rho was computed.

During case–case study hormone receptor-positive vs. hormone receptor-negative postmenopausal breast cancer patients by quartiles of serum steroid concentration were compared. The hormone receptor negative cases were categorized into four classes according to hormone levels. Receptor-positive/receptor-negative ratio was calculated belong to the four serum hormone concentration ranges. Odds ratios (ORs) were computed taking the lowest category of hormone receptor-negative cases as reference (Begg and Zhang 1994). ORs with 95% CIs and P_trend_ are presented by quartile limits of serum parameters.

Chi-square test was used (with Yates’ correction for continuity) for the investigation of independence of numerical variables and the determination of linear trends (P_trend_) among the groups classified by tumor receptor status.

## Results

### Selection of case subjects

IDC patients (1,042 cases) were categorized according to histological grade (HG), stadium (St) and ER, PR, HER2 receptor status (Table 1).

**Table 1:** Categorization of the subjected primary breast cancer patients suffering invasive ductal carcinoma (IDC) (total number: 1,042) according to histological grade (HG), stadium (St) and receptor status

Factor	Number
*IDC histological grade (HG) (IDC total number: 1,042)*	
HG 1	244
HG 2	425
HG 3	373
*IDC stadium (St) (IDC total number: 1,042)*	
1	489
2	336
2a	103
2b	43
3	47
3a	15
3b	2
3c	7
*Receptor status (IDC total number: 1,042)*	
ER+	852
ER−	190
PR+	709
PR−	333
ER+/PR+	703
ER−/PR−	184
ER+/PR−	149
ER−/PR+	6
HER2+	123
HER2−	949
HER2+/ER+/PR+	33
HER2−/ER+/PR+	670
HER2−/ER+/PR−	125
HER2+/ER−/PR−	65
HER2−/ER−/PR−	119

*ER* estrogen receptor, *PR* progesterone receptor, *HER2* human epidermal growth factor receptor 2.

We classified the cases according to their joint status of hormone and HER2 receptors. For the case–case study we defined the following tumor subtypes: ER+ (852 cases) vs. ER− (190 cases); PR+ (709 cases) vs. PR− (333 cases); ER+/PR+ (703 cases) vs. ER−/PR− (184 cases); ER+/PR− (149 cases) vs. ER−/PR− (184 cases) and HER2+ (123 cases) vs. HER2− (949 cases) (Table 1). In addition, further subgroups were also established according to the combined presence/absence of ER, PR and HER2. These subgroups were as follows: HER2+/ER+/PR+ (33 cases) vs. HER2−/ER+/PR+ (670 cases); HER2+/ER−/PR− (65 cases) vs. HER2−/ER−/PR− (119 cases) and HER2−/ER+/PR+ (670 cases) vs. HER2−/ER+/PR− (125 cases) (Table 1).

### Serum hormone levels

Hormone measurements were completed for all 1,042 cases except E2. Until 2005, the IMMUNOTECH RIA kit had been used for serum E2 measurements, which had low sensitivity in the range of <40 pmol l^−1^. The values of E2 concentration measured by the two different kits significantly differ from each other. Therefore, the measurements done with IMMUNOTECH RIA kit were excluded from the analysis (392 subjects).

Later on the serum E2 concentration was measured with an ultra-sensitive RIA kit in 650 (62%) patients. The average E2 value was 60.53 pmol l^−1^ (95% CI of the mean was 54.97–66.09), therefore this ultra-sensitive kit is appropriate for the detection of low serum E2 level in the majority of postmenopausal women. Only these data were used in the statistical analysis.

**Fig. 1:**
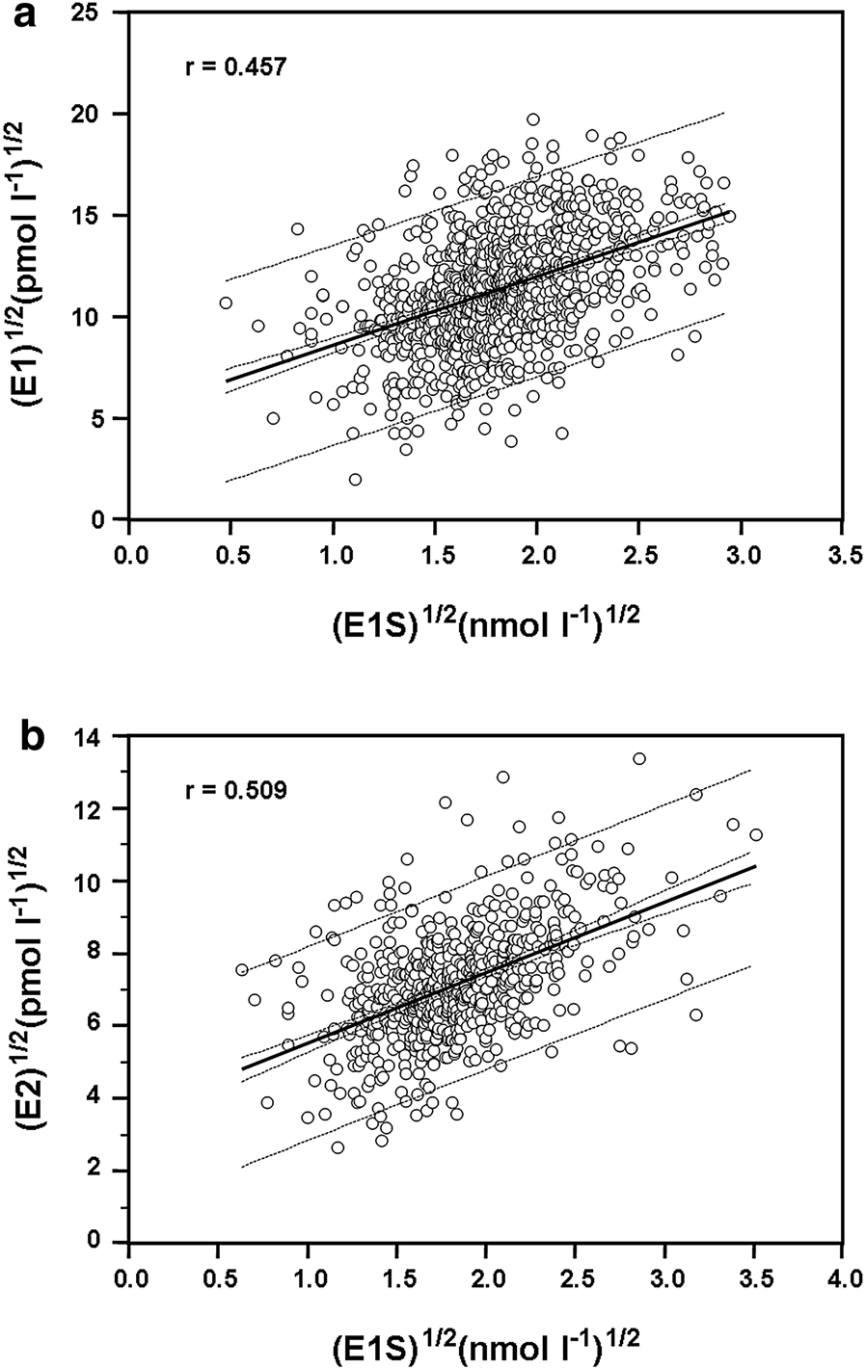
Correlation between the normal distributed serum concentration of E1S ^½^ (nmol l^−1^)^½^ and E1 ^½^ (pmol l^−1^)^½^ (**a**) and E1S ^½^ (nmol l^−1^)^½^ and E2 ^½^ (pmol l^−1^)^½^ (**b**). Serum E1 (966 cases) and E2 (631 cases) levels are significantly correlated with E1S (r = 0.457, p < 0.0001 and r = 0.509, p < 0.0001), respectively. Correlation coefficient (r), 95% confidence and 95% prediction intervals are shown on the figure. *E1* estrone, *E1S* estrone sulfate, *E2* estradiol, *CI* confidence interval.

**Fig. 2:**
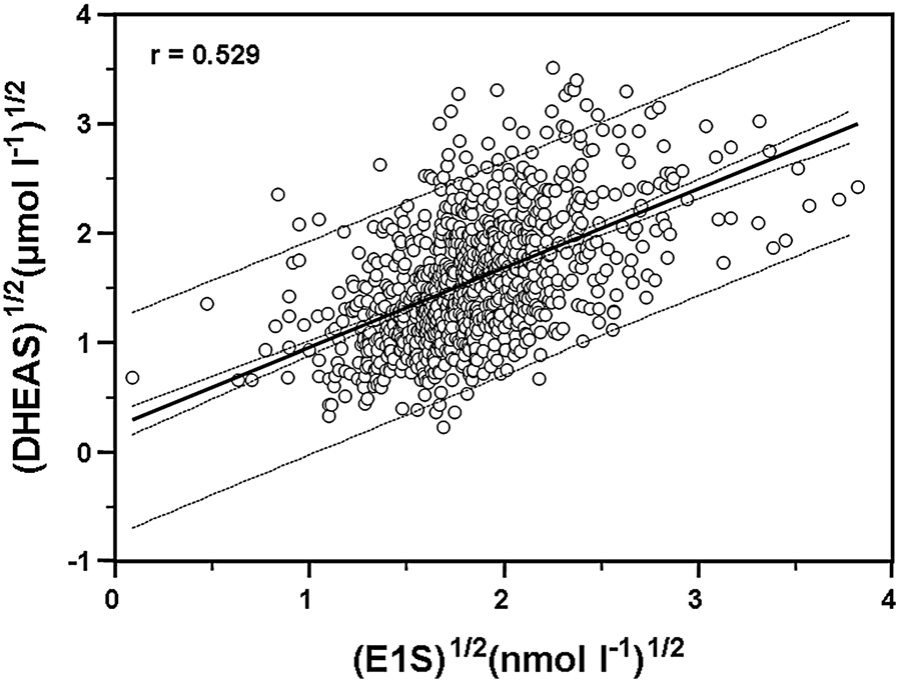
Correlation between the normal distributed serum concentration of DHEAS ^½^ (µmol l^−1^) ^½^ and E1S^½^ (nmol l^−1^)^½^. Sulfoconjugated steroids, measured in primary postmenopausal IDC breast cancer patients (989 cases) prior to surgical intervention, correlated significantly with each other (r = 0.529, p < 0.0001). Correlation coefficient (r), 95% confidence and 95% prediction intervals are shown on the figure. *E1S* estrone sulfate, *DHEAS* dehydroepiandrosterone sulfate, *CI* confidence interval.

Serum concentrations of the steroid hormones correlated significantly with each other. DHEAS significantly correlated with DHEA r = 0.704 (p < 0.0001), TE/SHBG ratio r = 0.441 (p < 0.0001) and AD rho = 0.391 (p < 0.0001) (data are not shown). Serum E1 and E2 levels strongly correlated with DHEAS (r = 0.543, p < 0.0001 and r = 0.463, p < 0.0001) (data are not shown) and with E1S, the major substrate for STS (r = 0.457, p < 0.0001 and r = 0.509, p < 0.0001), respectively (Fig. 1a, b). E1 and E2 correlated significantly with each other r = 0.518 (p < 0.0001) and with androgens (AD, free TE) (data are not shown). The association between E1 and AD, as well as E2 and TE/SHBG, was r = 0.415 (p < 0.0001) and r = 0.433 (p < 0.0001), respectively (data are not shown). The serum levels of sulfoconjugated steroids (DHEAS, E1S) also correlated significantly with each other r = 0.529 (p < 0.0001) (Fig. 2).

### Case–case study

To assess the impact of receptor status on serum steroid hormone levels, case–case comparisons and Chi-square test were used (OR, 95% CI, P_trend_). IDC patients (1,042 cases) were categorized into different classes based on hormone receptor status as written earlier.

In ER+ vs. ER− tumors the alteration of ORs were significant only in case of E1 level (OR = 1.663, p = 0.0026, P_trend_ = 0.0101) (Table 2).

**Table 2:** Case–case comparison of receptor-positive vs. receptor-negative postmenopausal IDC breast cancer patients by quartiles of serum steroid concentrations

Receptor status	Case–case study, odds ratio								
	E2 (pmol I^−1^)	E2/SHBG	E1 (pmol l^−1^)	E1S (pmol l^−1^)	E1S/E1	AD (nmol l^−1^)	TE (nmol l^−1^)	TE/SHBG	BMI (kg/m^2^)
(ER+)/(ER−)	0.82	1.09	1.66**	1.26	0.72	1.24	1.50	1.20	1.12
(PR+)/(PR−)	1.09	1.36	1.5*	1.08	0.65*	1.52*	1.36	1.39	1.66*
(ER+/PR+)/(ER−/PR−)	0.84	1.18	1.77*	1.23	0.66	1.31	1.56	1.29	1.70*
(HER2+)/(HER2−)	1.32	1.99	0.53*	2.44**	3.12***	0.81	0.99	1.04	0.48*
(HER2+/ER−/PR−)/(HER2−/ER−/PR−)	0.25	1.00	0.56	1.08	2.08	0.71	1.13	1.33	0.46
(HER2−/ER+/PR+)/(HER2−/ER+/PR−)	1.67	1.99	1.38	1.07	0.58	1.51	1.11	1.43	1.89
*(HER2+/ER+/PR+)/(HER2−/ER−/PR−)*	*2.00*	9.00*	*1.50*	17.00**	6.00*	*2.14*	11.60*	*2.25*	*0.42*
*(HER2+/ER+/PR+)/(HER2−/ER+/PR+)*	*6.94*	*7.00*	*0.83*	17.00*	31.19*	*1.22*	*1.66*	*2.25*	*nd*

Odds ratios in the fourth quartile are shown. Rows in italics present comparisons where number of cases is small therefore these results are only informative. Further investigations are needed.

*ER* estrogen receptor, *PR* progesterone receptor, *HER2* human epidermal growth factor receptor 2, *E1* estrone, *E1S* estrone sulfate, *E2* estradiol, *TE* testosterone, *AD* androstenedione, *SHBG* sex hormone binding globulin, *BMI* body mass index, *OR* odds ratio, *nd* no data.

Statistical significance: * p < 0.05, ** p < 0.01, *** p < 0.001.

**Fig. 3:**
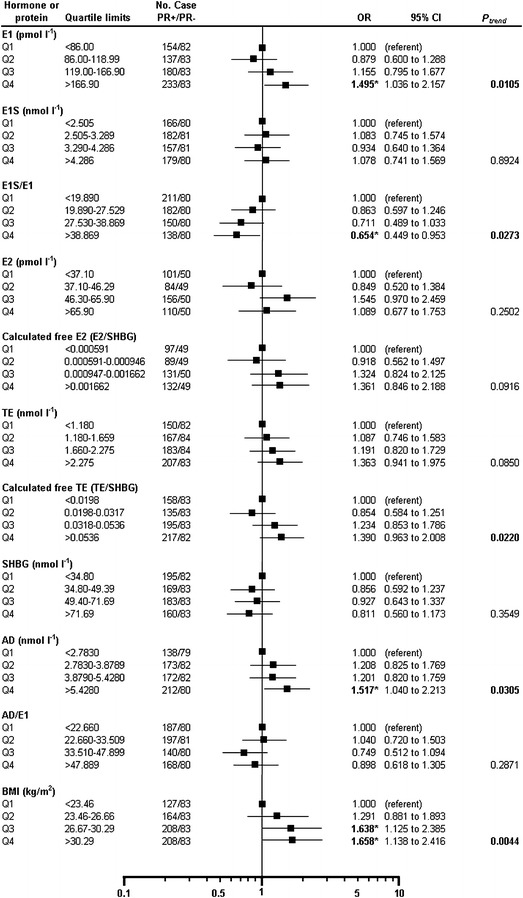
Case–case comparison of progesterone receptor-positive (PR+) vs. progesterone receptor-negative (PR−) postmenopausal IDC breast cancer patients by quartiles of serum steroid concentration. Odds ratios (ORs) were computed taking the lowest category of hormone receptor-negative cases as reference. ORs with 95% CIs and P_trend_ are presented by quartile limits of serum parameters. *Black squares* show ORs in quartiles (Q1–Q4), and the* horizontal lines* show 95% CIs. ORs are shown on a log scale. Chi-square test was used for determination of linear trends (P_trend_) among the groups classified by tumor receptor status. *ER* estrogen receptor, *PR* progesterone receptor, *E1* estrone, *E1S* estrone sulfate, *E2* estradiol, *TE* testosterone, *AD* androstenedione, *SHBG* sex hormone binding globulin, *BMI* body mass index, *OR* odds ratio, *CI* confidence interval. Statistics: *p < 0.05.

Our results showed that E1 levels were significantly elevated in the fourth quartile of PR+ vs. PR− tumors (OR = 1.495, p = 0.0317; P_trend_ = 0.0105). The same association was observed in case of serum AD levels (OR = 1.517, p = 0.0305; P_trend_ = 0.0394). Due to association between E1S and E1 levels we examined the association between E1S/E1 ratio and hormone receptor status (PR+ vs. PR−). E1S/E1 ratio significantly decreased (OR = 0.654, p = 0.0273) and a significant trend was also present (P_trend_ = 0.0151). There was no significant difference in serum E2, TE SHBG concentrations and E2/SHBG ratio. The TE/SHBG ratio showed only a significant trend (P_trend_ = 0.022). In case of BMI, we found a significant elevation in the third (OR = 1.638, p = 0.0487) and fourth quartiles (OR = 1.658, p = 0.0409) with a significant trend (P_trend_ = 0.0044), as well (Fig. 3).

In ER+/PR+ vs. ER−/PR− cases, the same trend (P_trend_ = 0.0078) for E1 was observed in the fourth quartile as described in ER+ vs. ER− cases (OR = 1.766, p = 0.0158) (Table 2). Likewise PR+ vs. PR− cases, BMI presented the same tendency in the third (OR = 1.639, p = 0.0379) and in the fourth quartiles (OR = 1.697, p = 0.0261) with a positive trend (P_trend_ = 0.0244).

**Fig. 4:**
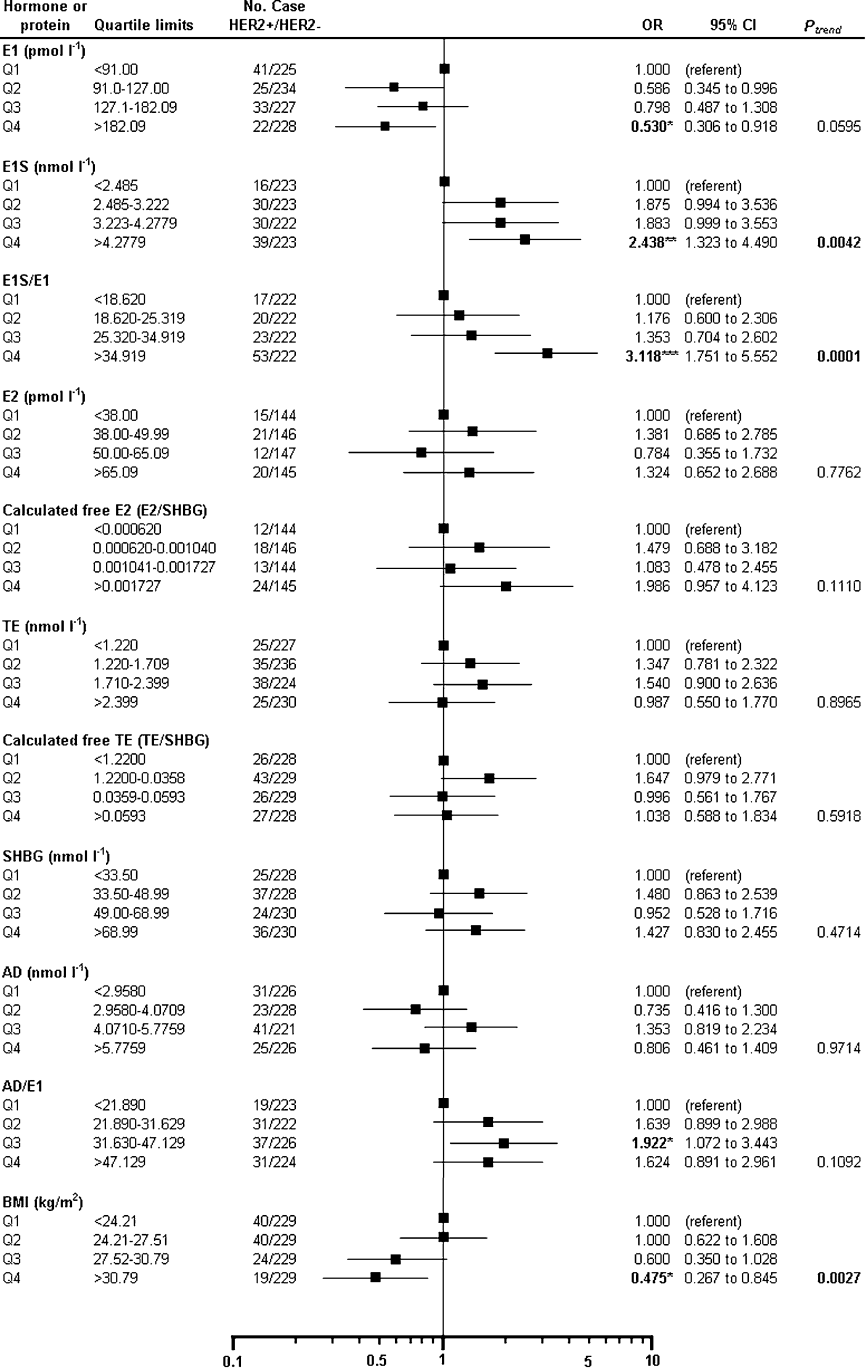
Case–case comparison of HER2-positive vs. HER2-negative postmenopausal IDC breast cancer patients by quartiles of serum steroid concentrations. Odds ratios (ORs) were computed taking the lowest category of hormone receptor-negative cases as reference. ORs with 95% CIs and P_trend_ are presented by quartile limits of serum parameters. *Black squares* show ORs in quartiles (Q1–Q4), and the *horizontal lines* show 95% CIs. ORs are presented on a log scale. Chi-square test was used for determination of linear trends (P_trend_) among the groups classified by tumor receptor status. *HER2* human epidermal growth factor receptor 2, *E1* estrone, *E1S* estrone sulfate, *E2* estradiol, *TE* testosterone, *AD* androstenedione, *SHBG* sex hormone binding globulin, *BMI* body mass index, *OR* odds ratio, *CI* confidence interval. Statistics: *p < 0.05, **p < 0.01, ***p < 0.001.

This is the first study that investigated associations between serum sexual hormones and HER2 status of the tumors in invasive ductal breast cancer. HER2 overexpression (HER2+ vs. HER2−) was assessed with significantly decreased E1 levels (OR = 0.530, p = 0.0234). Serum E1S levels showed a significant elevation and positive trend (P_trend_ = 0.0066) in the fourth (OR = 2.438, p = 0.0042) quartiles in HER2-positive cases. The ratio of E1S/E1 was increased significantly (OR = 3.118, p = 0.0001) with a positive trend (P_trend_ < 0.0001). In addition, AD/E1 ratio also showed significant elevation (OR = 1.922, p = 0.0282) in the third quartile, but the trend was not seen. BMI significantly decreased in the fourth quartile (OR = 0.475, p = 0.0113) and the trend was also significant (P_trend_ = 0.0027) (Fig. 4).

Contrary to ER+ vs. ER− and ER+/PR+ vs. ER−/PR− subgroups in HER2−/ER+/PR+ vs. HER2−/ER+/PR− cases serum E2, E2/SHBG and E1S/E1 ratios showed significant or nearly significant trend. OR of E2 (OR = 1.667) and E2/SHBG (OR = 1.992) increased in the fourth quartile; with positive trend (P_trend_ = 0.0544, P_trend_ = 0.0257). Similar to PR+ vs. PR− cases, OR of E1S/E1 decreased to 0.582 in the fourth quartile and the trend was nearly significant (P_trend_ = 0.0530). OR of BMI increased in the fourth quartile (OR = 1.887) with a nearly significant trend (P_trend_ = 0.0538) (Table 2).

HER2+/ER−/PR− vs. HER2−/ER−/PR− cases did not show any significant changes. Interestingly, E1S/E1 ratio elevated in the fourth quartile (OR = 2.083) with an almost significant positive trend (P_trend_ = 0.0724) (Table 2). In addition, AD/E1 (OR = 2.750, p = 0.0387) and TE/SHBG (OR = 2.667, p = 0.0363) ratios showed significant elevation in the second quartile.

Taking account of hormone receptor (HR) status next to HER2 positivity we found the greatest variances in the ORs of hormones and SHBG during comparison of HR− and HR+ subgroups (HER2+/ER+/PR+ vs. HER2−/ER+/PR+ and HER2+/ER+/PR+ vs. HER2−/ER−/PR− comparisons). It should be noted that because of the small number of triple positive cases further investigations are needed.

In the triple-positive vs. HER2−/ER+/PR+ cases the OR of E2, E2/SHBG, E1S and E1S/E1 significantly elevated in the fourth quartile with a significant trend except E2 where trend was not found. The OR of TE increased in the third quartile, while OR of TE/SHBG already elevated in the second quartile, but none of them showed a trend (Table 2).

The most interesting associations were found in the triple-positive vs. triple-negative cases. Significant elevation was found in case of E2/SHBG, E1S, E1S/E1 and TE in the fourth quartile with a significant trend. Serum AD also showed significantly elevated OR in the third quartile but the trend was not seen. The OR of BMI decreased remarkably without any trend (Table 2).

In ER+/PR− vs. ER−/PR− and HER2−/ER+/PR+ vs. HER2−/ER−/PR− cases we did not find any association between receptor status and serum steroid hormone levels.

## Discussion

The strength of our study compared with previous reports is the large number of IDC cases (1,042 patients) which enables us to evaluate the association between serum parameters and receptor status not only in HR-positive, but HER2-positive and HR-negative cases.

In our study the serum level of steroid hormones, hormone-precursors and SHBG were measured prior to surgical intervention in postmenopausal women with primary breast cancer. The associations between E1 and E1S and between E2 and E1S and between DHEAS and E1 indicate the importance of circulating E1S and DHEAS as peripheral estrogen pools in serum (Labrie 2015).

After menopause, E2 plasma level decreases by 90% (Russo and Russo 2006) and the primary estrogen is E1. Others have already reported that serum E2 level is not a reliable risk predictor for postmenopausal women. This may be due to methodological issues as E2 levels are generally around the level of detection in menopause; therefore, E1 levels are preferred (Miyoshi et al. 2003a). In patients with diagnosed breast cancer our results for E2, E1 and E1S are consistent with literary data (Würtz et al. 2012). Based on our case–case studies the serum E1 concentration and E1S/E1 ratio associated tumor HR and HER2 status. Of note, high E1 level and low E1S/E1 ratio associate with HR-positivity, particularly PR+ and ER+/PR+ cases. Regarding HER2, decreased E1 level and elevated E1S/E1 ratio were measured in HER2-positive cases.

Our results also support the pivotal role of STS in peripheral cells (e.g. platelets and lymphocytes) (Bonser et al. 2000; Garrido et al. 2012) and tissues (i.e. normal breast and breast carcinoma cells). Presumably, serum E1S influences intratumoral estrogen biosynthesis through STS pathway. STS enzyme activity was detected in the great majority of breast carcinomas (Evans et al. 1994). In postmenopausal women the sulfatase pathway is more dominant then the aromatase route. The uptake and efflux of sulfoconjugated steroid hormones are mediated by transport proteins (Ugele et al. 2003). It was shown that organic anion transport protein 2B1 (OATP2B1) is responsible for the uptake of E1S in the cell (Ugele et al. 2003). OATP2B1 protein is strongly expressed in the epithelial cells in invasive ductal carcinomas of the breast. Its expression level correlated with the grade and stage of the disease (Al Sarakbi et al. 2006). Several studies reported that the expression of STS in tumor cells might imply the progression of the tumor and indicate a poor clinical outcome (Evans et al. 1994; Utsumi et al. 1999; Miyoshi et al. 2003b; Suzuki et al. 2009). In addition, increased STS expression has also been associated with clinical resistance to endocrine therapies (Chanplakorn et al. 2010) and higher histological grades (Al Sarakbi et al. 2006; Geisler et al. 2011).

Interestingly in triple receptor-positive cases (HER2+/ER+/PR+) the elevated steroid hormone concentrations and the significantly increased E1S/E1 ratio compared with HER2−/ER+/PR+ or triple-negative cases were associated with HR-positivity and HER2-positivity, respectively. This phenomenon might be due to molecular cross-talk between ER and the HER2 pathways (Dowsett et al. 2008; Tripathy et al. 2013; Mehta and Tripathy 2014). Several studies indicated that the estrogen-mediated activation of G-protein coupled ER1 (GPER1) was associated with several rapid cellular signaling events including activation of epidermal growth factor receptor (EGFR) (Filardo et al. 2006; Ignatov et al. 2010; Ignatov et al. 2011; Jiang et al. 2013). According to De Francesco et al. (2014) estrogenic GPER signaling is able to trigger hypoxia-inducible factor 1A (HIF1A)-dependent vascular endothelial growth factor (VEGF) expression that supports angiogenesis and progression in breast cancer (De Francesco et al. 2014).

Another possible explanation is that estrogens promote the growth, stromalization and angiogenesis of an ER-negative breast cancer cell line (Gupta and Kuperwasser 2006; Gupta et al. 2007). These studies suggest that E2 can act as a potent metastasis-promoter in ER-negative tumors by a novel mechanism involving the host microenvironment.

Best to our knowledge we are the first who demonstrated that elevated E1S concentration and E1S/E1 ratio may be linked with HER2-positive tumors and possibly may indicate an impact of STS pathway. Because of the small number of HER2+/ER+/PR+ cases further investigations are needed to verify this hypothesis.

Our findings that serum E1 level and BMI are significantly elevated in PR+ and ER+/PR+ cases is in line with literary data and supports the role of aromatase route (Cauley et al. 1989). In HER2+ cases a decreased E1 level is associated with a significantly decreased BMI. In postmenopausal breast cancer patients obesity-associated higher estrogen levels might be explain with that aromatization is the major source of estrogens in contrast to an ovarian source in premenopausal women. An increasing volume of adipose tissue in obesity is associated with an increase in total body aromatase activity (Goodwin 2013). The surrounding adipose tissue has an influence to the steroid biosynthesis in the tumor itself. Our hypothesis is that instead of aromatase, STS pathway will be preferred in HER2+ cases.

These results raise the notion that macro-environmental concentrations and conversion of conjugated-unconjugated E1 in peripheral tissue and aromatization of AD and TE to E1 and E2 in adipose tissue might influence the micro-environmental (normal breast) and intrinsic (breast tumor) biosynthesis of estrogens. Our result draws attention to the importance of STS pathways. Measurement of serum E1 and E1S concentrations, and their ratio, and the assessment of tumor receptor status might support the selection of the appropriate therapy, and the individualization of regime.

Therefore, the measurement of serum estrone and estrone sulfate concentrations prior to surgical intervention in postmenopausal primary breast cancer patients might be of benefit.

## Abbreviations

AD: androstenedione
BMI: body mass index
CI: confidence interval
DHEA: dehydroepiandrosterone
DHEAS: dehydroepiandrosterone sulfate
EGFR: epidermal growth factor receptor
E2: estradiol
ER: estrogen receptor
E1: estrone
E1S: estrone sulfate
FISH: fluorescence in situ hybridization
GPER1: G-protein coupled ER1
HRT: hormone replacement therapy
HR: hormone receptor
HER2: human epidermal growth factor receptor 2
HIF1A: hypoxia-inducible factor 1A
IHC: immunohistochemical
IDC: invasive ductal carcinoma
DCIS: invasive ductal carcinoma in situ
ILC: invasive lobular carcinoma
OATP2B1: organic anion transport protein 2B1
OR: odds ratio
PR: progesterone receptor
SHBG: sex hormone binding globulin
STS: steroid sulfatase
TE: testosterone
VEGF: vascular endothelial growth factor
